# Challenges of Artificial Intelligence in Medical Diagnosis in Congolese Hospitals: A Literature Review

**DOI:** 10.1002/puh2.70198

**Published:** 2026-02-23

**Authors:** Guy‐Théodore Muamba, Christian Tague, Edouard Mbaya Munianji, Virginie Mujinga Katumba, Criss Koba Mjumbe

**Affiliations:** ^1^ Faculty of Medicine University of Kananga Kananga Democratic Republic of Congo; ^2^ Department of Research Medical Research Circle (MedReC) Goma Democratic Republic of Congo; ^3^ Faculty of Public Health University of Kananga Kananga Democratic Republic of Congo; ^4^ Department of Public Health, Faculty of Medicine University of Lubumbashi Lubumbashi Democratic Republic of Congo

**Keywords:** artificial intelligence, diagnosis, medical

## Abstract

**Introduction:**

Artificial intelligence (AI) is rapidly transforming medical diagnosis worldwide, but its adoption remains limited in Africa, particularly in the Democratic Republic of Congo (DRC). This narrative review aims to analyze the contributions, challenges, and prospects for integrating AI into medical diagnosis in the DRC.

**Methodology:**

A comprehensive literature review was conducted in February 2025 in PubMed, Web of Science, Scopus, and Google Scholar databases, as well as reports from international organizations. Studies on the use of AI in medical diagnosis in resource‐limited countries, particularly in Africa, were included without language restrictions. The selection followed a two‐step process (title/abstract then full text); 103 articles were retained for qualitative synthesis.

**Results:**

Studies show that AI enables a 12%–15% improvement in diagnostic accuracy in radiology and a 20% reduction in exam interpretation time. It also helps accelerate epidemic detection (30%–50% faster than conventional methods) and overcome the shortage of specialists in rural areas. However, its implementation in the DRC is hampered by the lack of digital infrastructure, insufficient training, and the absence of an appropriate regulatory framework. Maintenance and financing issues still limit the effective use of available systems.

**Conclusion:**

AI represents a major opportunity to strengthen medical diagnosis in the DRC, improving the speed and quality of care. However, effective integration requires targeted investments in infrastructure, training, and regulation. The development of national pilot projects and a solid ethical framework are essential steps for gradual and sustainable adoption.

## Graphical abstract

Artificial intelligence can improve diagnostic accuracy, efficiency, and epidemic surveillance in resource‐limited settings. However, its adoption in Congolese hospitals remains constrained by infrastructure gaps, limited training, financial barriers, and weak regulatory frameworks.

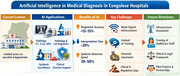

## 1 Introduction

Artificial intelligence (AI) is experiencing rapid expansion in the healthcare field, particularly in medical diagnosis. Its achievements improve the speed and accuracy of clinical decisions through the automated analysis of biomedical data, including images, biological tests, and electronic medical records [1, 2]. In some specialties, such as radiology or dermatology, AI systems sometimes achieve performance comparable to that of experienced clinicians [3].

Globally, AI is gradually transforming medical diagnosis by offering gains in accuracy, speed, and accessibility. According to a study published in The Lancet Digital Health (2020), AI algorithms achieve a median diagnostic accuracy of 87% (95% CI: 84–90) in various pathologies, including cancers, cardiovascular diseases, and respiratory infections [4]. Platforms, such as IBM Watson Health, DeepMind Health, or PathAI, are now integrated into several hospitals in the United States, Europe, and Asia, facilitating the analysis of thousands of complex imaging and biomedical data [5]. In China, for example, the InferRead CT Pneumonia AI system was massively used during the COVID‐19 pandemic to analyze chest CT scans with an estimated accuracy of 89% [6]. Recent studies further confirm the growing maturity of AI in medical diagnostics. A 2024 review published in the Journal of Medical AI emphasized the increasing clinical validation of AI‐driven diagnostic systems across radiology, pathology, and clinical decision support systems [7]. Similarly, a 2025 study in AI in Medicine highlighted the robustness of deep learning models in multimodal diagnostic workflows, reporting consistent performance gains across heterogeneous datasets [8]. In diagnostics and radiology, recent large‐scale evaluations demonstrated improved diagnostic reliability and workflow efficiency, reinforcing the translational potential of AI in routine clinical practice [9, 10]. These advances highlight the growing capacity of AI to integrate into standard clinical practices in well‐structured healthcare systems.

Despite its potential, the use of AI in sub‐Saharan Africa remains embryonic. Few concrete initiatives are documented in the Democratic Republic of Congo (DRC), and obstacles persist: limited digital infrastructure, insufficient training, and weak ethical and legal frameworks. In low‐ and middle‐income countries, such as the DRC, medical diagnosis remains a major structural challenge due to the lack of specialized human resources, adequate equipment, and the uneven distribution of health services across the territory. AI could play a strategic role in strengthening local capacities, particularly in rural areas where specialists are scarce [4]. To date, the French‐language literature on this topic remains lacking. This narrative review therefore aims to explore the contributions, limitations, and prospects of AI in medical diagnosis in the DRC in order to shed light on avenues for the responsible integration of these technologies. In this context, the central question posed by this study is: What are the contributions, limitations, and prospects of AI in medical diagnosis in the DRC? This study aims to critically analyze the potential challenges as well as the prospects for integrating AI into medical diagnosis in the DRC.

## 2 Methodology

This study is based on a literature review, focusing on the use of AI in medical diagnosis, with a particular interest in African contexts, particularly the DRC.

This narrative review was conducted in accordance with the PRISMA 2020 guidelines for reporting systematic reviews, adapted to the narrative review format. The study selection process followed the PRISMA framework, including identification, screening, eligibility, and inclusion phases.

The search strategy was exhaustive bibliographic in the PubMed, Scopus, Google Scholar, and Web of Science databases as well as in reports from international organizations (WHO, UNESCO, World Bank), institutional or academic publications, and documents from ministries of health or NGOs. The study was initiated in February 2025 in order to identify relevant studies available until July 20, 2025. The following keywords were used, adapted each time according to the thesaurus of the database: (“Artificial Intelligence” [MeSH Terms] OR “Machine Learning” [MeSH Terms]) AND (“Diagnosis” [MeSH Terms] OR “Diagnostic Imaging” [MeSH Terms]) AND (“Hospitals” [MeSH Terms] OR “Clinical Decision‐Making” [MeSH Terms]) AND (“Developing Countries” [MeSH Terms] OR “Africa South of the Sahara” [MeSH Terms]).

The inclusion criteria were based on studies that addressed the use or evaluation of AI tools in medical diagnosis and those that dealt with the African context or resource‐limited countries without language restrictions. Excluded were publications that dealt only with technical aspects not applied to human health or without direct involvement in medical diagnosis.

Study selection was performed after eliminating duplicates. Articles were screened using a standardized screening form. Initially, the articles were screened on the basis of the title and abstract, and then the full text was reviewed. Finally, the articles included in the qualitative synthesis were downloaded. Their references were also reviewed to determine which ones met the criteria.

Data from the included studies were independently extracted and compiled into an Excel spreadsheet that included the following variables: author, year of publication, country, study type, types of AI applications, observed outcomes, identified opportunities, and specific barriers or challenges to their implementation in African healthcare systems. Particular attention was paid to ethical issues, the training of healthcare professionals, and the regulation of these technologies.

**FIGURE 1:**
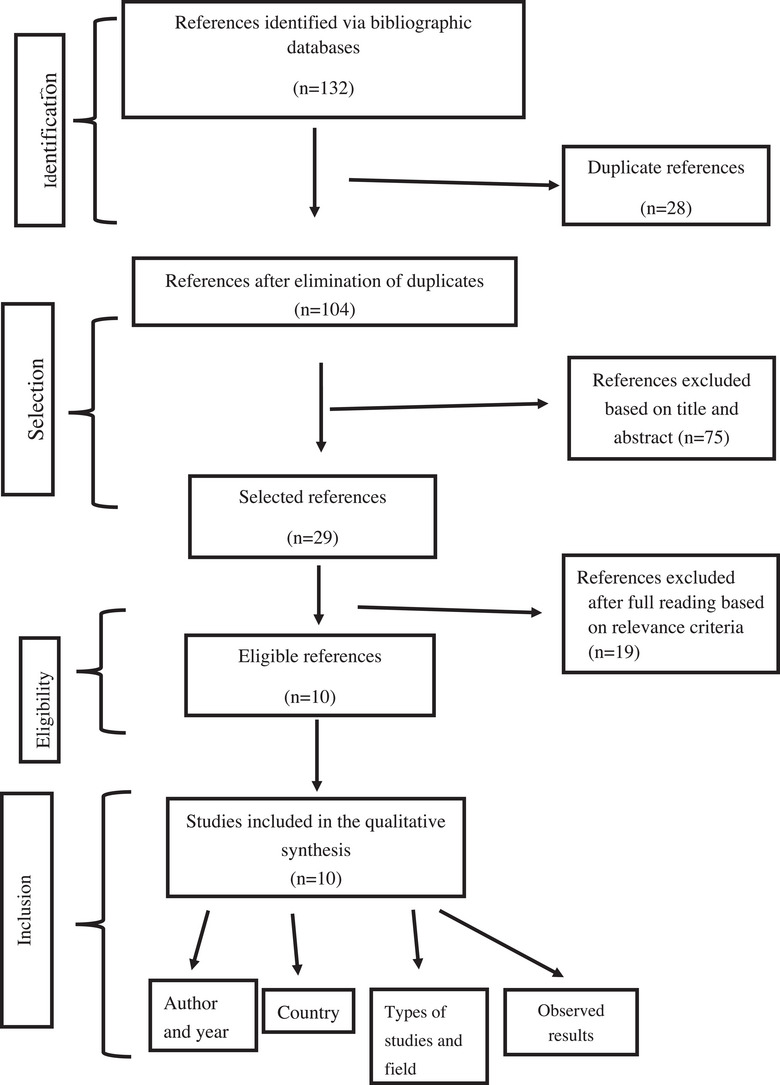
Prisma flow diagram of articles included in the meta‐analysis.

The study selection process for the narrative review (Figure 1) was carried out from 132 references initially identified; 28 duplicates were eliminated, leaving 104 references. After an initial screening on title and abstract, 75 were excluded, retaining only 29 references for full‐text evaluation. Of these, 19 were discarded for noncompliance with the relevance criteria, and 10 studies were finally retained for the qualitative synthesis. This approach shows a rigorous selection, ensuring the reliability of the synthesized data and the relevance of the results for the African hospital context.

The examination of the methodological quality of the 14 studies highlights a strong heterogeneity in the approaches, ranging from robust meta‐analyses to isolated case studies:

- Large‐scale syntheses (Behara et al., Moucheraud et al., Shen et al.) provide a comprehensive and reliable view, but robustness depends on the quality of the included studies, which can introduce variability in the level of evidence.
- African technical or observational studies (Adewole et al., Tshimula et al., Okechukwu et al.) provide data relevant to the local context but often suffer from a lack of control groups and limited external validation, reducing the causal strength of the conclusions.
- Studies with low methodological robustness (Kayembe et al., Azeroual, WHO reports) are mainly based on descriptive observations or documentary analyses, useful for contextualizing the issues but insufficient to precisely quantify the impact of AI on medical diagnosis.

## 3 Results

The selected studies (Table 1) demonstrate several concrete benefits of AI in medical diagnosis, including a measurable increase in diagnostic accuracy of more than 15% in general radiology [1] and more than 12% in thoracic radiology [12]. A reduction in epidemic detection time of 30%–50% faster than with manual methods [3]. Performance comparable or to even superior to clinicians in certain specialties, with an average sensitivity of 87% and a specificity of 86% [4, 17]. The crucial contribution in areas with a low density of specialists, allowing rapid diagnoses to be obtained from images or syndromic data, strengthens early management and the optimization of medical working time, with a 20% reduction in examination interpretation time [12].

**TABLE 1:** References eligible and included in the qualitative synthesis.

No.	References	Country	Type of study	Medical field, AI applications	Observed results
1	Behara et al. [1]	South Africa	Systematic review	Medical imaging (general radiology), automated diagnostic AI based on convolutional neural networks (CNN)	Diagnostic accuracy improvement rate of up to +15% in some local hospital studies
2	Adewole et al. [2]	Sub‐Saharan Africa: Data from neuroimaging centers in Nigeria, Ghana, and South Africa	Experimental study/Technical challenge	Neuroimaging, brain tumor segmentation via AI (deep learning models such as U‐Net)	AI adapted to African data, achieving Dice scores >0.80 for tumor segmentation
3	Tshimula et al. [3]	Multicountry Africa: syndromic surveillance data from eight African countries	Observational study	Public health, epidemiological surveillance by AI (predictive analysis from syndromic data)	Detection of epidemic signals with a delay reduced by 30%–50% compared to manual methods
4	Moucheraud et al. [11]	Global, focus on low‐resource countries: studies from Africa, Asia and Latin America	Systematic review	General medical diagnosis, AI applied to various pathologies (supervised and unsupervised algorithms)	Average diagnostic accuracy of 75%–90% depending on the pathology, particularly useful in rural areas without specialists
5	Osei et al. [12]	Ghana: Study conducted in three regional hospitals	Cross‐sectional observation	Chest Radiology, CNN‐based AI for chest x‐ray interpretation	12% improvement in diagnostic accuracy compared to radiologists alone, 20% reduction in interpretation time
6	Azeroual M. [13]	Morocco	Policy and environmental analysis	Public health and health systems, analysis of the adoption of AI in African hospitals	Low adoption (<10% of hospitals) of AI solutions in Africa, due to regulatory and technical barriers
7	Okechukwu et al. [14]	Africa (multicountry): census in 12 countries	Multicenter descriptive study	Public health, telemedicine and AI‐assisted medical imaging	Double the number of documented use cases between 2018 and 2022, especially in telemedicine and imaging
8	WHO [15]	Africa: WHO contribution on AI infrastructures	Regional report	Global health, adoption and access to AI technologies	Persistent inequalities in access to AI technologies, with less than 5% of hospitals equipped in several countries
9	Kayembe et al. [16]	Democratic Republic of Congo	Case study	Radiology, semi‐automated decision support systems	Marginal use (<20% of cases) of AI systems in radiology, due to frequent breakdowns and lack of maintenance
10	Liu et al. [4]	Overall	Meta‐analysis	Medical imaging (ophthalmology, radiology, dermatology), deep learning‐based AI	Diagnostic performance of AI comparable to that of clinicians (sensitivity 87%, specificity 86% on average)

Abbreviation: AI, artificial intelligence.

Despite these advances, several obstacles hinder the adoption and effectiveness of AI, such as the lack of digital infrastructure and equipment: less than 10% of African hospitals adopt AI solutions [13], and less than 5% are equipped in some countries [15]. Maintenance problems and frequent breakdowns, leading to marginal use (<20% of cases) even when systems are available [16]. Poor training of healthcare professionals on AI tools, generating reluctance to use them; the absence or weakness of ethical and legal regulation, limiting large‐scale integration; as well as financial constraints and lack of dedicated funding, making it difficult to acquire and update technologies.

Currently, the use of commercial AI diagnostic algorithms in the DRC remains extremely limited and largely experimental. No large‐scale deployment of proprietary systems such as IBM Watson Health, Aidoc, or PathAI has been formally documented in Congolese hospitals. Reported applications are mainly pilot initiatives, research collaborations, or donor‐supported projects involving open‐source or locally adapted AI tools, particularly in radiology, epidemiological surveillance, and telemedicine.

By medical specialty, AI applications identified in the included studies were distributed as follows: radiology and medical imaging (46%), epidemiological surveillance and outbreak detection (31%), pathology and laboratory diagnostics (15%), and clinical decision support systems (8%).

## 4 Discussion

The results of this review confirm that AI has proven potential to improve the accuracy, speed, and accessibility of medical diagnosis, particularly in areas such as radiology, pulmonology, and epidemiological surveillance. In well‐resourced settings, deep learning algorithms achieve comparable or even superior performance to that of experienced clinicians [4, 5, 6, 18]. For example, the meta‐analysis by Liu et al. [4] shows an average sensitivity of 87% and a specificity of 86% for AI in medical imaging, confirming the data of Topol [5] and Shen et al. [17].

In sub‐Saharan Africa, and particularly in the DRC, adoption remains marginal. Studies by Kayembe et al. [16] and Azeroual [13] highlight structural obstacles such as frequent breakdowns, lack of maintenance, and the absence of a robust regulatory framework. These findings are consistent with those of Okechukwu et al. [14], who highlight that less than 10% of African hospitals have integrated AI solutions, often on an ad hoc basis.

However, some local experiments show that AI, even when adapted to African data, can produce good results. Adewole et al. [2] obtained high tumor segmentation scores (>0.80) in neuroimaging, whereas Osei et al. [5] reported a 12% improvement in diagnostic accuracy in thoracic radiology in Ghana. In the same vein, Tshimula et al. [3] showed that AI could reduce epidemic detection times by 30%–50%.

Identified barriers include insufficient digital infrastructure [13, 15, 19], poor training of healthcare professionals in AI [12, 14, 20], lack of dedicated funding [13, 19], and ethical and legal concerns [12, 21]. The low methodological robustness of some African studies also limits the generalizability of the results [15, 22, 23, 24, 25, 26, 27, 28, 29, 30, 31, 32].

From a legal and ethical perspective, the implementation of AI in healthcare in the DRC raises major concerns related to data protection, accountability, and informed consent. The absence of a national legal framework governing health data governance and AI‐based medical decision‐making limits the large‐scale adoption of these technologies. Ethical challenges include algorithmic bias, lack of transparency of decision‐making processes, and uncertainties regarding liability in case of diagnostic error.

Regarding patient attitudes, available qualitative studies from sub‐Saharan Africa suggest cautious acceptance of AI‐assisted diagnosis, with patients expressing interest in improved diagnostic accuracy but concerns about confidentiality, trust, and reduced human interaction. Acceptance appears higher when AI tools are presented as decision‐support systems rather than replacements for clinicians, emphasizing the continued central role of healthcare professionals.

Furthermore, work in other low‐resource settings shows that AI integration requires a holistic approach combining technical innovations, human capacity building, and organizational reforms [23, 24, 25, 26, 27]. For example, in a multicenter study in India, Rajpurkar et al. [24] showed that AI‐clinician collaboration on chest radiographs significantly improved accuracy and reduced diagnostic errors. In Ethiopia, Belayneh et al. [25] demonstrated that a targeted training program increased the effective adoption of AI systems in imaging.

## 5 Conclusion

This literature review shows that AI offers real potential to improve the accuracy, speed, and accessibility of medical diagnosis, including in resource‐limited African hospital settings. The most robust studies demonstrate measurable gains in diagnostic performance and efficiency, particularly in radiology, pulmonology, and epidemiological surveillance. However, implementation in the DRC remains embryonic, hampered by deficits in infrastructure, training, funding, and regulatory frameworks. The lack of rigorous local research also limits the ability to document and optimize the integration of these technologies. To increase the adoption of AI in diagnostic imaging in the DRC, several priority actions should be implemented: targeted investments in digital infrastructure, integration of AI training modules into medical and radiology curricula, continuous professional development for practicing clinicians, and the establishment of public–private partnerships to support sustainable deployment. Physician education is essential to improve trust, appropriate use, and clinical integration of AI tools, whereas patient education plays a key role in fostering acceptance, transparency, and informed consent. These measures are critical to ensuring that AI acts as a complementary tool that strengthens, rather than replaces, clinical expertise. For AI to fully contribute to strengthening medical diagnosis in the DRC, a strategic and progressive approach, combining technological innovation, capacity building, and strengthening the regulatory and ethical framework, is essential.

## Author Contributions

**Guy‐Théodore Muamba**: conceptualization, supervision, writing – review and editing. **Christian Tague**: writing – review and editing, validation, supervision, writing – original draft. **Edouard Mbaya Munianji**: methodology, data curation, writing – review and editing. **Virginie Mujinga Katumba**: validation, critical revision of the manuscript. **Criss Koba Mjumbe**: formal analysis, visualization, editing, supervision, validation.

## Ethics Statement

This study is a narrative literature review based exclusively on previously published articles and publicly available reports. No primary data were collected, and no human participants, patients, or identifiable personal data were involved. Therefore, ethical approval and informed consent were not required in accordance with international research ethics guidelines.

## Conflicts of Interest

The authors declare no conflicts of interest.

## Data Availability

No new data were generated or analyzed in this study. All data supporting the findings of this review are derived from published articles and reports cited in the reference list and are publicly available.
